# Design of High-Precision Parallel AWG Demodulation System

**DOI:** 10.3390/mi14091662

**Published:** 2023-08-25

**Authors:** Yunjing Jiao, Qijing Lin, Kun Yao, Na Zhao, Dan Xian, Fuzheng Zhang, Qingzhi Meng, Bian Tian, Zhuangde Jiang

**Affiliations:** 1 State Key Laboratory of Mechanical Manufacturing Systems Engineering, Xi’an Jiaotong University, Xi’an 710049, China; 2 School of Mechanical and Manufacturing Engineering, Xiamen Institute of Technology, Xiamen 361021, China; 3 Shandong Laboratory of Advanced Materials and Green Manufacturing at Yantai, Yantai 264000, China; 4 Bright Stone Industrial Technology Research Institute, Yantai 265503, China

**Keywords:** integrated optics, arrayed waveguide grating, stagger peak decouple

## Abstract

Here, we present a high-precision demodulation method that supports the arrayed waveguide grating (AWG) system, which includes a 1 × 8 AWG as the primary filter with a 0.5 nm channel spacing and a 1 × 4 AWG as the auxiliary filter with a 1 nm channel spacing. The high precision is achieved through an innovative method of decoupling three channels, involving two adjacent channels of the primary filter and one channel of the secondary auxiliary filter. Simulation results show that the AWGs have a good transmission spectrum with crosstalk below −24.8 dB, non-uniformities below 0.8 dB, insertion loss below −3.7 dB, 3 dB bandwidth of 0.25 nm, and 10 dB bandwidth of 0.43 nm. The interrogation precision can reach 8 pm, with a dynamic range of 0.4 nm, corresponding to a single FBG.

## 1. Introduction

Spectral analysis is a crucial element of fiber optic sensing technology. The physical quantity carried by the spectrum can be obtained by analyzing the amplitude, phase, wavelength, and other parameters of the light wave. Spectrum demodulation is an integral part of spectral analysis. Generally, a traditional spectrometer is used to perform spectrum demodulation, but its large size, high price, and complex structure make it difficult to carry and limit its application. Integrated optics devices will have a more attractive prospect if they can maintain good performance of spectrum demodulation compared with a traditional spectrometer [1]. Recently, the demodulation system based on photonic integrated circuit (PIC) technology has gained more attention with the rapid development of PIC [2,3], which uses planar optical waveguides to connect and integrate optical emission, coupling, transmission, reception, and processing devices on a chip. Therefore, the demodulation system has more obvious advantages in size, weight, and power consumption.

Arrayed waveguide grating (AWG) is generally used as the core optical splitter in the demodulation system based on PIC technology. There are several key parameters of AWG in the optical structure design stage, including diffraction order, number of arrayed waveguides, length of free propagation region, spacing of waveguides, and others [4]. In addition, performance parameters such as crosstalk, insertion loss, and loss uniformity also need to be considered when further optimizing [5]. There is a certain relationship in the AWG demodulation system where the dynamic range, wavelength resolution, and interrogation precision restrict each other. It is significant improvement to the performance of the AWG interrogation system if it could maintain the high interrogation precision without reducing the wavelength resolution and dynamic range.

The present research of AWG demodulation systems tends to improve the resolution and dynamic range at the same time. Trita proposed an AWG with 50 nm of spectrum range, and the resolution was 2.5 pm, but the accuracy was about 40 pm from the results [6]; Li studied an ultrasmall AWG system with 0.8 nm channel spacing with the interrogation performance not mentioned [7] and an AWG-based demodulation system [8] with a high precision of ±10 pm, yet it did not mention the dynamic range. However, it could be observed from the graphic results that the dynamic range is smaller than the channel spacing of 0.3 nm. Weng studied a multi-channel AWG, with the resolution of 1.27 pm, the dynamic range of 1.2 nm, and the precision of 20.6 pm [9] and an AWG with the precision of 24.59 pm and the dynamic range of less than 1.3 nm [10]. Li proposed an AWG with the precision of 31.4 nm and the dynamic range of 0.8 nm. The research works show that the dynamic range and interrogation precision are mutually restricted and cannot be optimized at the same time, that is, the high interrogation accuracy can only be achieved in a small dynamic range. There is a method to improve the interrogation accuracy by increasing the spectral overlap of the channels [11]. In addition, research shows that the AWG with denser channel spacing can improve the linearity of the demodulation system [12], e.g., in Ref. [6], the channel spacing of 0.18 nm leads to a large crosstalk, and the footprint is also large. In addition, the cascaded AWG is an effective research structure, which is generally composed of the two stages of the traditional AWG structure [13,14]. Han studied a cascaded AWG system with a resolution of 0.5 nm over 75 nm bandwidth [15]. However, this cascaded AWG is more beneficial to large bandwidths. In short, the research works present the difficulty to achieve the large dynamic range and high precision demodulation only through a single AWG in the demodulation system. Thus, it is significant to design an AWG demodulation system with high performance including relatively large dynamic range and high precision.

In this paper, we present a parallel AWG demodulation system that has denser channel spacing and larger spectral overlap. The parallel AWG system includes two AWGs to obtain larger dynamic range and high precision. An auxiliary 1 × 4 AWG is designed to form a demodulation system with a primary conventional 1 × 8 AWG. This AWG system can provide an innovative demodulation method, involving three channels of AWG. The three channels include two adjacent channels of 1 × 8 AWG and a channel of 1 × 4 AWG, which can perform a staggering peak decoupling method. We have simulated the transmission spectrum of AWG and FBG to ensure better performance of the demodulation system. We have analyzed the demodulation precision and dynamic range of the stagger peak decoupling method with different FBGs. Moreover, the comparison with traditional two-channels demodulation is carried out to verify the good demodulation performance.

## 2. Working Principle and Design

**Figure 1:**
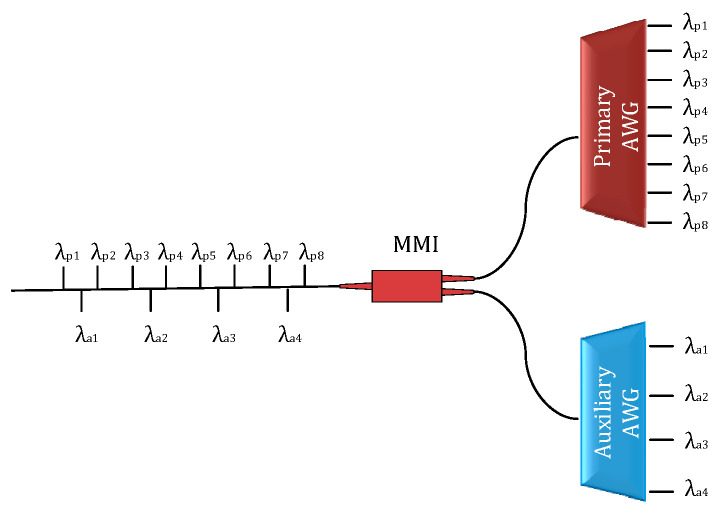
Schematic of the parallel AWG system.

**Figure 2:**
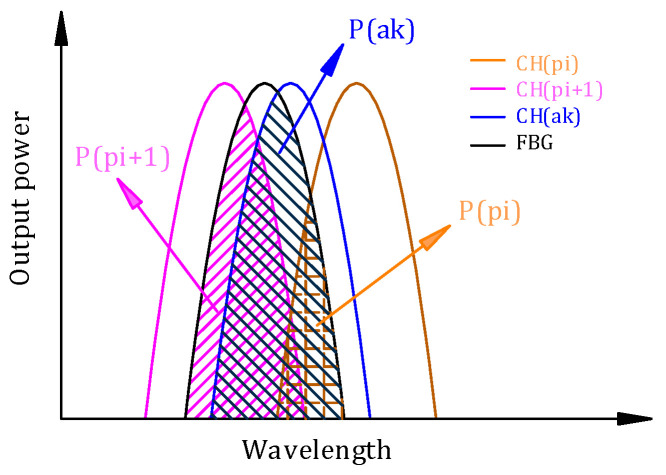
Schematic diagram of stagger peak decouples for FBG.

Figure 1 presents the theoretical structure of the parallel AWG system. The parallel AWG system consists of a 1 × 8 AWG as the primary filter and a 1 × 4 AWG as the auxiliary filter. A certain output channel wavelength of the auxiliary filter is the central value of two adjacent output channel wavelengths of the primary filter. Assuming that the light with peak wavelengths *λ*_p1_, *λ*_a1_, *λ*_p2_, *λ*_p3_, *λ*_a2_, *λ*_p4_, … *λ*_p8_ is input from the optical waveguide, after passing through the parallel AWG demodulation system, *λ*_p1_ to *λ*_p8_ will output from the primary filter and *λ*_a1_ to *λ*_a4_ will output from the auxiliary filter. In addition, *λ*_a1_ is the central value of *λ*_p1_, *λ*_p2_. A multimode interference (MMI) coupler, the splitting ratio of which is 50:50, could be used to connect the input waveguide of the 1 × 8 AWG and the 1 × 4 AWG to ensure the maximum power of the two AWGs. During the FBG demodulation, the FBG reflection light is first transmitted into the MMI coupler and then equally injected into two AWGs. The two beams could be assumed as identical. The FBG spectrum will output from two adjacent channels in the primary filter and one channel of the auxiliary filter. Then, the transmission spectrum of AWG overlaps with the FBG reflection spectrum to obtain the output spectra, that is, the spectra are convolved to gain the output power. P*_pi_* and P*_pi_*_+1_ are assumed to be the output optical power of two adjacent channels in the primary AWG and P*_ak_* is assumed to be output optical power of a certain channel in the auxiliary AWG (see Figure 2). When the FBG reflection spectrum alters due to the changes of related variables, the wavelength will move, and then the overlap with AWG will change. The output spectra of AWG also change next. It is assumed that the transmission coefficient and half-peak bandwidths are the same in each channel of AWG, then a linear function relationship will be exhibited between the FBG central wavelength (λ*_FBG_*) and the ratios of output optical power between AWG channels, as shown in Equations (10)–(12). Then, the FBG wavelength can be demodulated once the P*_pi_*, P*_pi_*_+1_, and P*_ak_* are detected.

**Figure 3:**
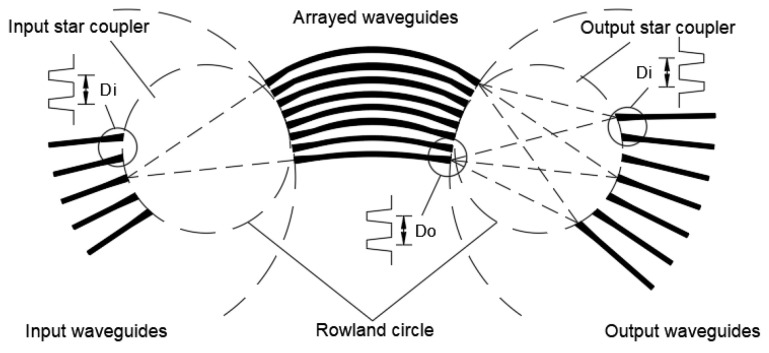
Schematic of single AWG.

As proof of principle, a parallel AWG system centered at λ*_c_* = 1550 nm was designed, as shown in Figure 1. Figure 3 shows the single AWG, which consists of input/output waveguides, two star couplers, and arrayed waveguides, where the star coupler is designed based on the Rowland circle.

When the incoming source is injected into the input waveguide, it is diffracted in the input star coupler and then transported into the arrayed waveguides, and the lights have the same phase currently. As the same path length differs with each other in adjacent arrayed waveguides, the lights of different wavelengths attain the same phase difference, which makes lights obtain a wavelength-dependent wave-front tilting when transmitting into the output star coupler. Then, the lights diffract again and interfere in the output star coupler. Finally, different wavelengths of light focus at certain positions and output from the corresponding output waveguide.

The main relationships of the AWG are shown below. We denote Δ*L* as the path length difference between each arrayed waveguide, *FSR* as the free spectral range of the AWG, *m* as the order of the array, and *R* as the diameter of the Rowland circle.

(1) $$\Delta L=m\lambda_{0}/n_{c}$$

(2) $$FSR=\lambda_{0}n_{c}/mn_{g}$$

(3) $$m\leq \lambda_{0}n_{c}/(N\Delta \lambda n_{g})$$

(4) $$R=n_{s}D_{i}D_{o}n_{c}/(m\Delta \lambda n_{g})$$

where *λ*_0_ represents the center operating wavelength, *n_c_* represents the effective index of the arrayed waveguide, *n_g_* represents the group index of the arrayed waveguide, Δ*λ* represents the spacing of the adjacent channel, *N* represents the maximal number of wavelength channels, *n_s_* represents the effective index of the slab waveguide, *D_i_* represents waveguide separation of input/output ports, and *D_o_* represents waveguide separation of array waveguides.

In order to further study the output spectral variation characteristics of AWG, the output transmission spectra of every channel and input are simulated. The transmission spectra can be assumed to be a Gaussian-like beam in every channel of AWG [9]. The equation for the channel (*pi*) of AWG can be depicted as:

(5) $$T_{AWG}(pi,\lambda )=T_{0}\exp\left[-4\ln2\frac{\left(\lambda -\lambda_{pi}{}^{2}\right)}{\Delta \lambda_{pi}{}^{2}}\right]$$

where *T*_0_ represents the attenuation coefficient of AWG, *λ_pi_* represents the center operating wavelength of channel *pi*, and Δ*λ_pi_* represents the half-peak bandwidths of channel *pi*.

The reflection spectrum of FBG can be supposed as Gaussian [9], and the equation of FBG will be:

(6) $$R_{FBG}(\lambda )=R_{0}\exp\left[-4\ln2\frac{\left(\lambda -\lambda_{B}{}^{2}\right)}{\Delta \lambda_{B}{}^{2}}\right]$$

where *R*_0_ represents the attenuation coefficient of FBG, *λ_B_* represents the central wavelength of FBG, and Δ*λ_B_* represents the half-peak bandwidth in FBG spectrum.

In the whole spectral region, the incoming source, FBG spectrum, and transmission spectrum of AWG are integrated to obtain the final output light power of AWG. Thus, the output optical power can be expressed as:

(7) $$P_{pi}=(1-L_{pi})\int_{0}^{\infty }I_{s}(\lambda )R_{B}(\lambda )T_{AWG}(pi,\lambda )d\lambda$$

(8) $$P_{pi+1}=(1-L_{pi+1})\int_{0}^{\infty }I_{s}(\lambda )R_{B}(\lambda )T_{AWG}(pi+1,\lambda )d\lambda$$

(9) $$P_{ak}=(1-L_{ak})\int_{0}^{\infty }I_{s}(\lambda )R_{B}(\lambda )T_{AWG}(ak,\lambda )d\lambda$$

where *P_pi_* and *P_pi_*_+1_ are the output optical power of the primary AWG’s adjacent channels (*pi*) and (*pi* + 1); *P_ak_* is the output optical power of the auxiliary AWG’s channel (*ak*); *L_pi_* and *L_pi_*_+1_ and *L_ak_* are the decay factors of the channel (*pi*) and channel (*pi* + 1) and channel (*ak*); *I_S_*(*λ*) is the optical power of the incoming source.

Then, we can obtain the FBG central interrogation wavelength through the ratio between output optical power of the AWG in each channel. The ratio relationships can be expressed as Equations (10)–(12). Equation (10) is the ratio relationship between the primary AWG adjacent channels. Equations (11) and (12) show the ratio relationship between the channels of the primary AWG and the auxiliary AWG. Here, we take channels p5 and p6 of the primary AWG and channel a3 of the auxiliary AWG as examples for easy understanding.

(10) $$\rho_{1}=\ln(\frac{P_{p5}}{P_{p6}})=\frac{8\ln(2)\Delta \lambda_{p5-p6}}{\Delta \lambda_{p6}{}^{2}+\Delta \lambda_{B}{}^{2}}\lambda_{B1}-\frac{4(\ln2)(\lambda_{p5}{}^{2}-\lambda_{p6}{}^{2})}{\Delta \lambda_{p6}{}^{2}+\Delta \lambda_{B}{}^{2}}$$

(11) $$\rho_{2}=\ln(\frac{P_{p5}}{P_{a3}})=\frac{8\ln(2)\Delta \lambda_{p5-a3}}{\Delta \lambda_{a3}{}^{2}+\Delta \lambda_{B}{}^{2}}\lambda_{B2}-\frac{4(\ln2)(\lambda_{p5}{}^{2}-\lambda_{a3}{}^{2})}{\Delta \lambda_{a3}{}^{2}+\Delta \lambda_{B}{}^{2}}$$

(12) $$\rho_{3}=\ln(\frac{P_{a3}}{P_{p6}})=\frac{8\ln(2)\Delta \lambda_{a3-p6}}{\Delta \lambda_{p6}{}^{2}+\Delta \lambda_{B}{}^{2}}\lambda_{B3}-\frac{4(\ln2)(\lambda_{a3}{}^{2}-\lambda_{p6}{}^{2})}{\Delta \lambda_{p6}{}^{2}+\Delta \lambda_{B}{}^{2}}$$

where *ρ*_1_ represents the power ratio between primary AWG channels p5 and p6, *ρ*_2_ represents the power ratio between primary AWG channel p5 and auxiliary AWG channel a3, and *ρ*_3_ represents the power ratio between auxiliary AWG channel a3 and primary AWG channel p6. Thus, we could obtain three central wavelengths of FBG through three ratios.

It is obvious that the reflected wavelength of FBG (*λ_B_*) has a linear relationship with the output optical power ratio between channels. Thus, we just need to measure the AWG output power (overlapping power) to demodulate the central wavelength of FBG. In addition, the wavelength interrogation precision could be improved compared with the traditional two channels due to the spectral overlap increasing.

## 3. Simulation and Results

**Figure 4:**
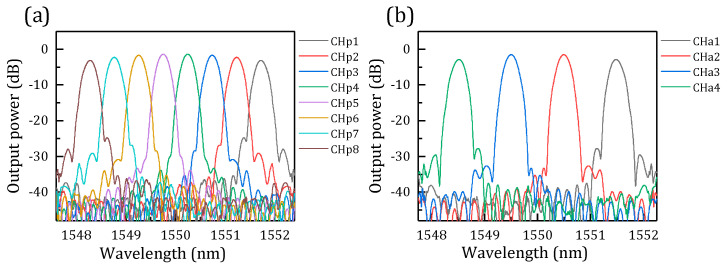
(**a**) Simulation results of primary 1 × 8 AWG. (**b**) Simulation results of auxiliary 1 × 4 AWG.

The parameters of the designed AWG are shown in Table 1. We designed a 1 × 8 primary AWG and a 1 × 4 auxiliary AWG to achieve a stagger peak decouple for FBG. For the two AWGs, the waveguide widths are all 0.45 um. From the simulation results, as shown in Figure 4a,b, it is found that insertion loss is smaller than −3.5 dB, insertion loss non-uniformities are below 0.8 dB, and the neighbor channel crosstalk of 1 × 8 AWG is below −24.8 dB. For the 1 × 4 AWG, the insertion loss is smaller than −3.7 dB, insertion loss non-uniformities are smaller than 0.8 dB, and the neighbor channel crosstalk is below −40 dB, which could be negligible.

**Figure 5:**
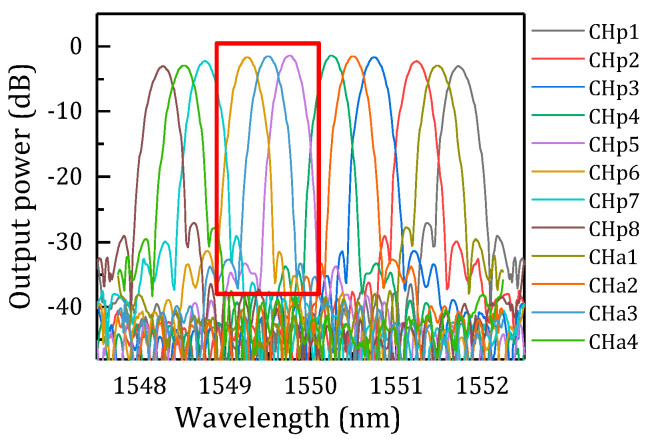
Simulation results of the combined spectrum.

We combined the two spectra, as shown in Figure 5, to clearly show the relative position of wavelength channels for the method of stagger peak decoupling. The output channel wavelength of the auxiliary filter is located between the two adjacent output channel wavelengths of the primary filter (channel a3 of the auxiliary AWG is located between the channels p5 and p6 of the primary AWG). The two AWG channels can be divided into three groups, for example, the channels p5 and p6 of the primary AWG and the channel a3 of the auxiliary AWG can become a group to perform the three-channel decouple, as shown in the red rectangular box. Currently, the center wavelength spacing between channel p5 and a3 is 0.25 nm, and so is the center wavelength spacing between channels 6 and 3. Thus, the channel spacing is reduced by 1/2 through the additional channel a3 without the smaller channel spacing design of AWG.

**Figure 6:**
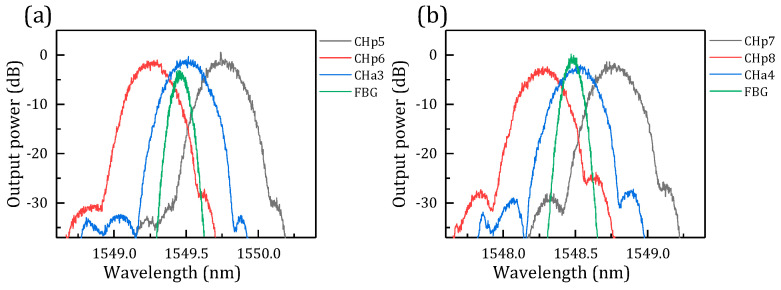
Simulation result of stagger peak decoupling (FBG central wavelength is 1549.64 nm). (**a**) The first group: channel a3 of the auxiliary AWG and channels p5 and p6 of the primary AWG. (**b**) The second group: channel a4 of the auxiliary AWG and channels p7 and p8 of the primary AWG.

Then, we simulated the position relationship and overlapped the AWG channels (the primary AWG channels p5 and p6 and the auxiliary AWG channel a3) and FBG, as shown in Figure 6. Two FBGs with different powers were taken into consideration to improve the credibility of the results.

From Figure 6a, FBG overlaps with channels p5, p6, and a3. Based on Equations (10)–(12), we calculated the overlapping power of each channel and FBG to obtain the central interrogation wavelength of FBG. We can find that the overlap of FBG and channels p5 and p6 is the traditional approach to FBG wavelength interrogation. The overlaps of FBG and channels p5 and a3 and channels p6 and a3 respectively show the increased stagger decoupling that the paper proposes.

**Figure 7:**
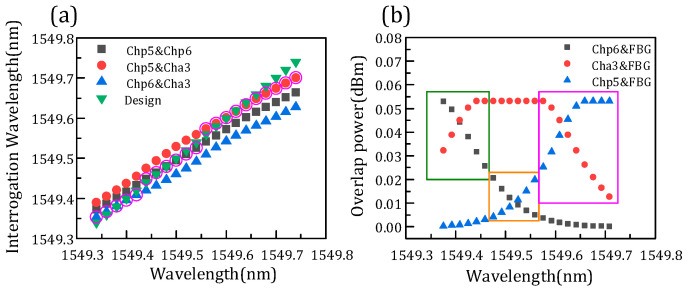
(a) FBG wavelength comparison of designed and interrogation. (b) Overlap power of channels and FBG.

**Figure 8:**
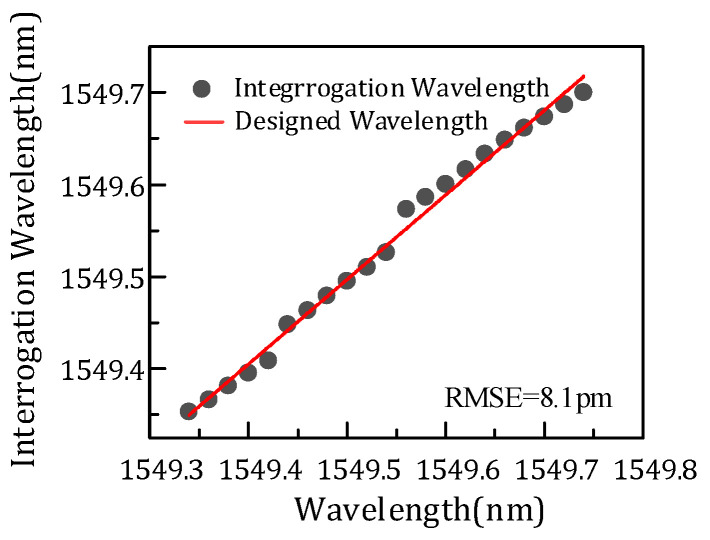
Interrogation results of the AWG demodulation system.

Figure 7a shows the comparison of three FBG interrogation wavelengths and the designed wavelength. As mentioned above, the FBG demodulation wavelength is related to the overlapping power. Figure 7b shows the overlapping power of channels and the FBG correspondent with the interrogation wavelength. The FBG central wavelength is designed from 1549.34–1549.74 nm. The optimal wavelength means that the three demodulation wavelengths differ from the design wavelength, and the one with the minimum difference is the optimal one. It is found that the optimum wavelength is related to the difference in overlap power. As shown in Figure 7a, the data circled in pink are the optimum wavelength. Meanwhile, the overlapping power of the three channels subtract from each other, and the two channels with minimum difference can demodulate the optimal wavelength, just corresponding to the result of Figure 7a. The part with a minimum difference is shown in the rectangle of Figure 7b. Thus, the FBG interrogation optimal wavelength is demodulated in channels p6 and a3, channels p6 and p5, and channels p5 and a3, with the increase of FBG wavelength in turn due to the different overlap power. Figure 8 presents the interrogation results of the AWG demodulation system, where the *x* axis is the designed wavelength and the *y* axis is the optimal interrogation wavelength corresponding to the data circled in pink in Figure 7a. In addition, the root-mean-square error is 8.1 pm by calculation. Therefore, the demodulation wavelength precision is 8.1 pm.

**Figure 9:**
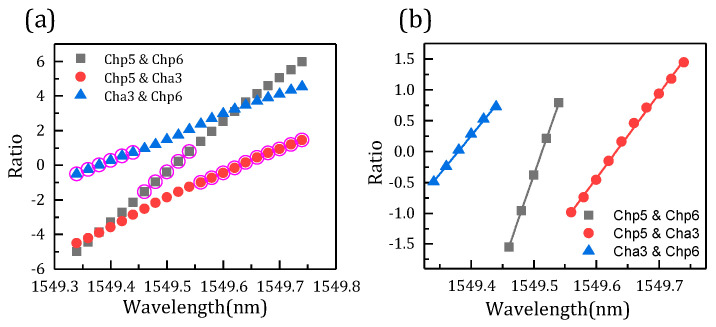
(a) The schematic diagram of the interrogation wavelength and power ratio. (b) Optimal ratio of AWG channels.

Figure 9a shows the relation between the FBG interrogation wavelength and optical power ratio. The data circled in pink are the optimal ratio corresponding to the optimal wavelength. From Figure 9b, the optimal ratio is fitted, and all the root-mean-square errors are less than 0.04 pm. Therefore, the optimal ratio can well predict the FBG demodulation wavelength. 

**Figure 10:**
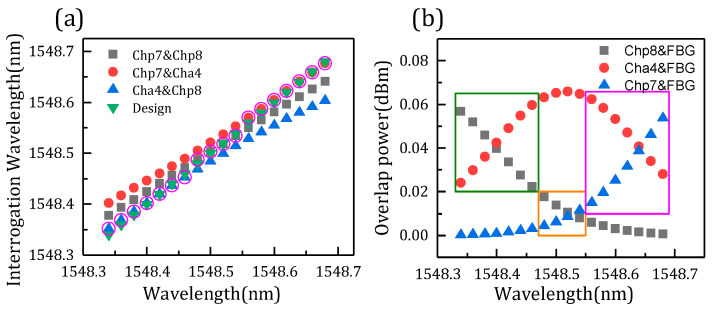
(**a**) FBG wavelength comparison of designed and interrogation. (**b**) Overlap power of channels and FBG.

**Figure 11:**
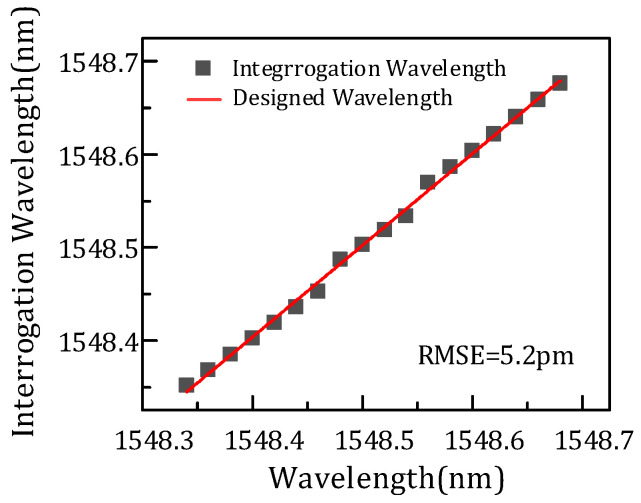
Interrogation results of the AWG demodulation system.

**Figure 12:**
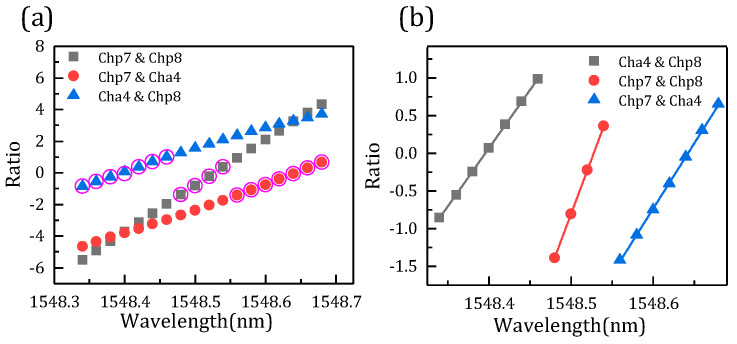
(**a**) The schematic diagram of the interrogation wavelength and power ratio. (**b**) Optimal ratio of AWG channels.

Another set of simulation analysis is performed for further verification, including channels p7, p8, and a4. Moreover, the power of the FBG central wavelength is changed. Similarly, the comparison of three FBG interrogation wavelengths and the designed wavelength is shown in Figure 10a, and the data circled in pink are the optimum wavelength. Figure 10b shows the relationship between overlap power and wavelength, and the optimal value in the rectangle also corresponds to Figure 10a. Figure 11 presents the interrogation results of the AWG demodulation system, where the *x* axis is the designed wavelength and the *y* axis is the optimal interrogation wavelength corresponding to the data circled in pink in Figure 12a. In addition, the root-mean-square error is 5.2 pm by calculation. Therefore, the demodulation wavelength precision is 5.2 pm. Figure 12a shows the relation between the FBG interrogation wavelength and optical power ratio. The data circled in pink are the optimal ratio corresponding to the optimal wavelength. From Figure 12b, the optimal ratio is fitted, and all the root-mean-square errors are less than 0.005 pm. The optimal ratio is consistent with the above results and can well predict the demodulation wavelength. In addition, the demodulating dynamic range in this paper is about 0.4 nm, which is limited by the channel spacing of the two AWGs.

Additionally, a comparation is presented between the proposed stagger peak decoupling method and the traditional approach (see Table 2). The proposed stagger peak interrogation method has better performance, its interrogation precision is about 8 pm, and its dynamic range is about 0.4 nm. Considering this result, we can well predict the interrogation wavelength for the AWG interrogator.

Thus, the FBG central wavelength with high precision would be decoupled by the two channels with minimum difference of overlap power. Generally, the two channels are in the primary AWG and the auxiliary AWG. Thus, the FBG optimal wavelength is decided as long as the AWG output power is detected in the stagger peak decoupling method. The wavelength precision could reach 8 pm, and the dynamic range is 0.4 nm in each interrogation.

## 4. Discussion

We proposed a parallel configuration AWG system with 1550 nm as the center wavelength based on SOI for FBG sensors. The AWG system includes a 1 × 8 AWG and a 1 × 4 AWG to present a staggering peak decoupling method with three channels. The simulation results show that the AWG demodulation precision is related to the overlapping power of FBG and channels. We overlapped the spectrum of the FBG with the output spectrum of the three channels in AWG, and three overlap power values can be obtained. Then, the three values subtract from each other and a pattern can be found, where the smaller the overlap power difference, the higher the demodulation precision using the two overlap powers. Compared to the reported research, the FBG wavelength interrogation has a higher demodulation precision and larger dynamic range with the stagger peak decoupling method. Thus, the interrogation precision could reach 8 pm and the dynamic range of 0.4 nm. The power ratio of adjacent channels also has a good linear relationship with the interrogation wavelength. The interrogation wavelength for the AWG interrogator could be well predicted once the power is detected. For a better performance, the parallel AWG system with the stagger peak decoupling method is very appealing compared to a traditional AWG.

## Figures and Tables

**Table 1:** Parameters of designed 1 × 8 and 1 × 4 AWG.

	AWG (1 × 8)	AWG (1 × 4)
Diffraction order	180	199
Δ*L* (μm)	114	126
Number of arrayed waveguide channels	25	23
Free spectral range (nm)	5.0	4.5
Channel spacing (nm)	0.5	1
Diameter of the Rowland circle (μm)	41	37
3 dB bandwidth (nm)	0.25	0.20
10 dB bandwidth (nm)	0.43	0.40

**Table 2:** Comparison of different interrogation methods in research works.

Research Works	Precision	Resolution	Dynamic Range
Our proposed demodulation system	8 pm	/	0.4 nm
Demodulation system [6]	Less than 40 pm	2.5 pm	Less than 1.84 nm
Demodulation system [7]	10 pm	/	Less than 0.3 nm
Demodulation system [9]	20.60 pm	1.27 pm	1.2 nm
Demodulation system [10]	24.59 pm	1 pm	Less than 1.3 nm
Demodulation system [16]	31.4 pm	4 pm	0.8 nm

## Data Availability

The data presented in this study are available on request from the corresponding author. The data are not publicly available due to privacy.
